# TGF-β2-induced ANGPTL4 expression promotes tumor progression and osteoclast differentiation in giant cell tumor of bone

**DOI:** 10.18632/oncotarget.18629

**Published:** 2017-06-27

**Authors:** Bo Li, Ming Qian, Hao Cao, Qi Jia, Zhipeng Wu, Xinghai Yang, Tianyi Ma, Haifeng Wei, Tianrui Chen, Jianru Xiao

**Affiliations:** ^1^ Department of Bone Tumor Surgery, Changzheng Hospital, Second Military Medical University, Shanghai, China; ^2^ School of Life Science and Biopharmaceutics, Shenyang Pharmaceutical University, Shenyang, China; ^3^ Faculty of Psychology and Mental Health, Second Military Medical University, Shanghai, China

**Keywords:** giant cell tumor of bone, ANGPTL4, osteoclast differentiation, cell proliferation, angiogenesis

## Abstract

Although emerging studies have implicated that Aiopoietin-like 4 Protein (ANGPTL4) is related to the aggressiveness and metastasis of many tumors, the role of ANGPLT4 in giant cell tumor (GCT) of bone was rarely investigated. The mechanism of ANGPLT4 in tumor-induced osteoclastogenesis still remains unclear. In this study, we first demonstrated that ANGPTL4 was highly expressed in GCT compared to normal tissues, while we showed that TGF-β2 released by osteoclasts induced bone resorption could increase the expression of ANGPTL4 in GCTSCs. By using the luciferase reporter assay, we found that two downstreams of TGF-β2, Smad3 and Smad4, could directly activate the promoter of ANGPTL4, which might explain the mechanism of TGF-β2-induced ANGPLT4 expression. Moreover, knockout of ANGPTL4 by TALENs in GCTSCs inhibited tumor growth, angiogenesis and osteoclastogenesis in GCT *in vitro*. By using the chick chorio-allantoic membrane (CAM) models, we further showed that inhibition of ANGPTL4 suppressed tumor growth and giant cell formation *in vivo*. In addition, some new pathways involved in ANGPTL4 application were identified through microarray assay, which may partly explain the mechanism of ANGPTL4 in GCT. Taken together, our study for the first time identified the role of ANGPLT4 in GCT of bone, which may provide a new target for the diagnosis and treatment of GCT.

## INTRODUCTION

Giant cell tumor (GCT) of bone is a common primary bone tumor which typically occurs in the epiphyseal end of long bones and less locates in sacrum, pelvis and spine [1–3]. GCT predominantly occurs in the third and fourth decade of life with a slight predilection for females [4, 5]. The tumor is composed of osteoclast-like multinucleated giant cells, spindle-shaped stromal cells and mononuclear precursor cells [6]. Spindle-shaped stromal cells of GCT (GCTSCs) originated from mesenchymal stem cells in bone marrow play a neoplastic role in GCT [7, 8]. GCT is classified as a benign tumor with a potentially aggressive behavior, malignant transformation and metastasis are not common [9, 10]. Surgical resection in the treatment of GCT is recommended, but the overall recurrence rate from 18% to 60% remains high [11, 12]. Osteolysis formation is an essential feature of GCT [13], however, the mechanism of GCT is still not fully understood. Thus further understanding the biology of GCT is critically important.

Angiopoietin-like 4 Protein (ANGPTL4), a member of the angiopoietin-like family, is a secreted protein closely related to angiogenesis as well as lipid metabolism [14–16]. ANGPTL4 is mainly expressed in the liver, adipose tissue, and placenta as well as in ischemic tissues, and is up-regulated after fasting and hypoxia. It was identified as a paracrine or endocrine regulator of lipid metabolism and a target of peroxisome proliferators-activated receptors (PPARs) [16, 17]. In recent years emerging studies have implicated ANGPTL4's role in the aggressiveness and metastasis of tumors due to its function in angiogenesis, inflammation and hypoxia. The role of ANGPTL4 in lipid metabolism has been well investigated [16, 18], however, the role of ANGPTL4 in tumor biology remains largely undefined. Some reports suggested its tumor-suppressive function [19, 20], but others claimed its oncogenic function [21–23]. The controversial results drive the need of further research to address the role of ANGPLT4 in tumor progression.

In this study, ANGPTL4 was identified as a critical cytokine modulating the proliferation, angiogenesis and osteolysis of GCT. We first demonstrated that ANGPTL4 was highly expressed in GCT. Importantly, we showed that TGF-β2 released by osteoclasts induced bone resorption could increase the expression of ANGPTL4 in GCTSCs. Moreover, ANGPTL4 promoted GCTSC induced osteoclast proliferation and angiogenesis *in vitro* and *in vivo*. In addition, some new downstreams of ANGPTL4 were identified through microarray assay. Our results revealed the substantial role of ANGPLT4 in regulating the biological cycle formation of GCT-bone microenviroment.

## RESULTS

### ANGPTL4 is highly expressed in GCTSCs of GCT

**Figure 1:**
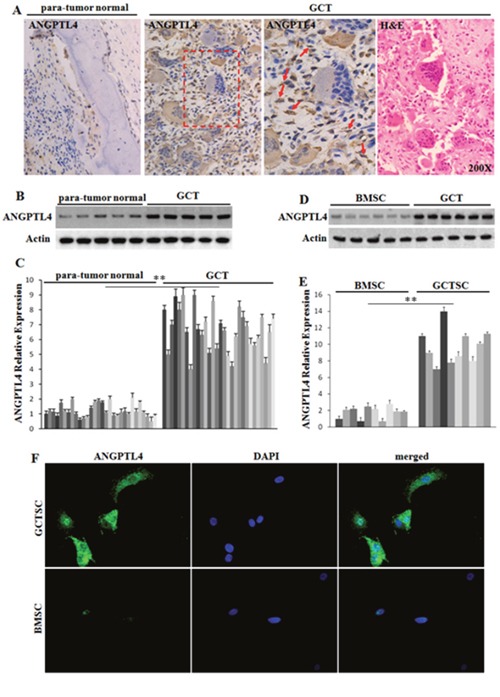
Expression profile of ANGPTL4 in GCT **(A)** immunolocalization and H&E staining of ANGPTL4 in human specimens of GCT and para-tumor normal bone formalin-fixed-paraffin-embedded tissues. ANGPTL4 was marked with red arrows. **(B** and **C)** western blot analysis and qRT-PCR analysis of ANGPTL4 expression level in GCT samples and para-tumor normal bone tissues. **(D** and **E)** western blot analysis and qRT-PCR analysis of ANGPTL4 expression levels of GCTSCs and BMSCs. **(F)** immunofluorescence staining of ANGPTL4 in GCTSCs and BMSCs, ** *p*<0.01.

IHC staining revealed that ANGPTL4 expression was elevated in GCT samples as compared with para-tumor normal bone tissues (Figure 1A). Furthermore, qRT-PCR and western blot assay also showed that the mRNA and protein levels of ANGPTL4 was higher in GCT samples than in para-tumor normal bone tissues (Figure 1B and 1C).

In order to figure out which cell is responsible for high ANGPTL4 expression in GCT, we compared the mRNA and protein expression levels of ANGPTL4 in GCTSCs with BMSCs. The results showed ANGPTL4 expression in GCTSCs were significantly higher than that in BMSCs (Figure 1D–1F). In addition, ELISA assay revealed that the levels of ANGPTL4 in serum of pre-operational patients were significantly higher than normal and post-operational cases (Supplementary Figure 1A). These results suggested that GCTSCs could contribute to the high level of ANGPTL4 in GCT.

### Expression of FOXO1, HIF-1α, PPAR-α and PPAR-γ in GCTSCs

**Figure 2:**
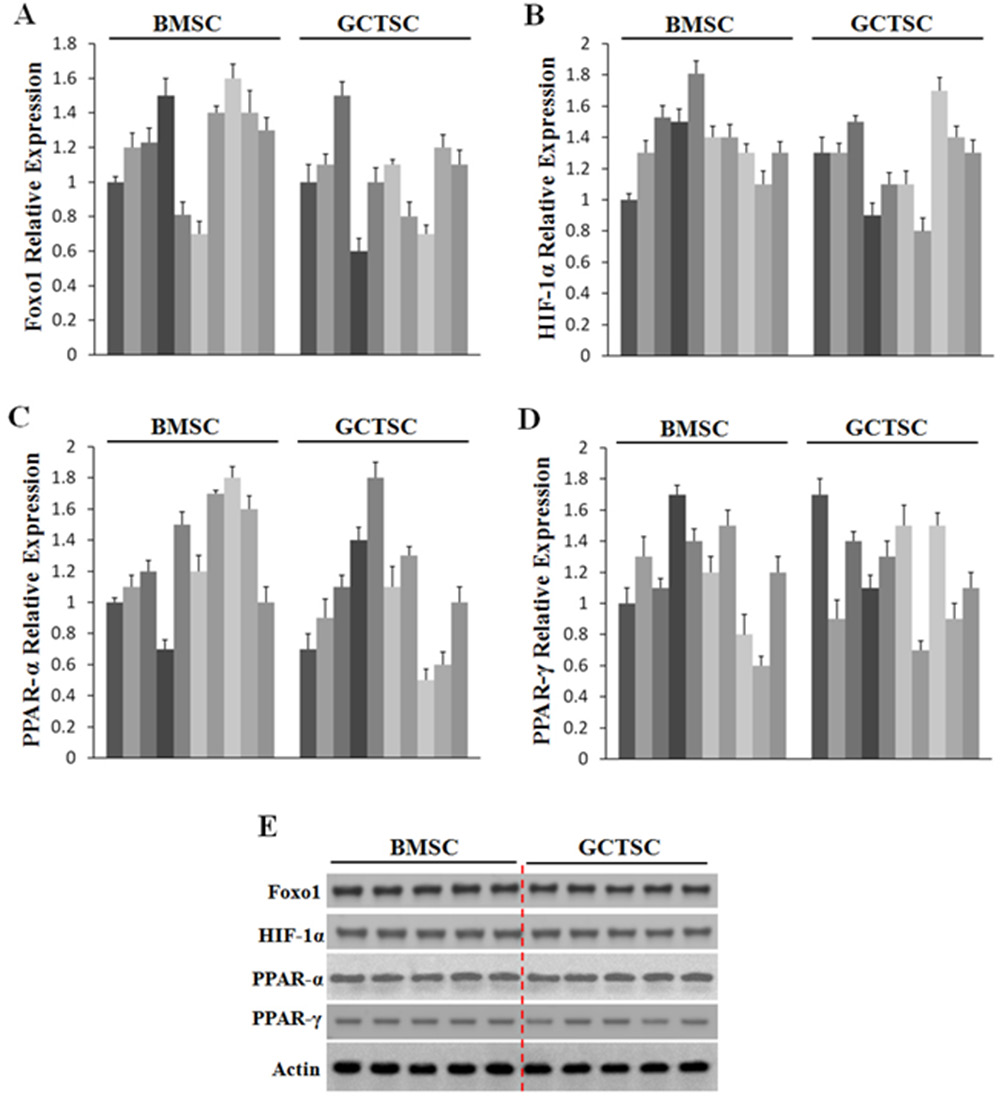
Expression profiles of Foxo1, HIF-1α, PPAR-α and PPAR-γ in GCT **(A-D)** qRT-PCR analysis of Foxo1, HIF-1α, PPAR-α and PPAR-γ mRNA levels in GCTSCs and BMSCs. **(E)** western blot analysis of Foxo1, HIF-1α, PPAR-α and PPAR-γ expression levels in GCTSCs and BMSCs.

According to the previous reports [27–30], the transcription factors (TFs), FOXO1, HIF-1α, PPAR-α and PPAR-γ, could enhance *ANGPTL4* expression through different mechanisms. In order to find out whether the expressions of these TFs were increased in GCTSCs, qRT-PCR and western blot analysis were conducted, but none of these factors changed between GCTSCs and BMSCs (Figure 2A–2E). These results indicated that the high level of ANGPTL4 may not be controlled by FOXO1, HIF-1α, PPAR-α or PPAR-γ in GCTSCs.

### Expression of ANGPTL4 enhanced by highly expressed TGF-β2 in GCTSCs

**Figure 3:**
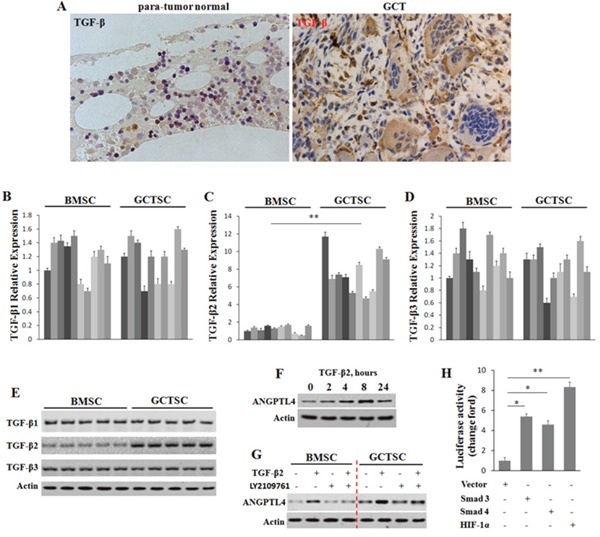
ANGPTL4 enhanced by highly expressed TGF-β2 in GCTSCs **(A)** immunolocalization of TGF-β in human specimens of GCT and para-tumor normal bone formalin-fixed-paraffin-embedded tissues. **(B-E)** mRNA and protein levels of TGF-β1, TGF-β2 and TGF-β3 were determined by qRT-PCR and western blot analysis. **(F)** GCTSCs were treated with TGF-β2, and expression levels of ANGPTL4 were analyzed by western blotting over times as indicated. Actin was used as a protein loading control. **(G)** GCTSCs and BMSCs were exposed to TGF-β2 and its inhibitor (LY2109761) for 8 hours. ANGPTL4 levels were analyzed by western blotting. **(H)** the effect of Smad3, Smad4 and HIF-1α on luciferase activity in GCTSCs, * *p*<0.05, ** *p*<0.01.

Previous studies showed that TGF-β can play a metabolic role in maintaining bone homeostasis and TGF-β bioavailability in the bone microenvironment is crucial in keeping the balance of bone remodeling [31]. In addition, a previous study was reported that TGF-β could prime breast tumors for lung metastasis through ANGPTL4 [21]. In order to figure out whether a high level of TGF-β induces the up-regulation of ANGPTL4 in GCT, we firstly performed the immunolocalization of TGF-β in human specimens of GCT and para-tumor normal bone tissues. The results showed that there was a significant enhancement of TGF-β in GCT (Figure 3A). Furthermore, to find out which ligands of TGF-β are important in this pathway, we compared the mRNA and protein levels of TGF-β1, TGF-β2 and TGF-β3 between GCTSCs and BMSCs. It was interesting to find out that only the expression of TGF-β2 was significantly higher compared to the other different ligands in GCTSCs (Figure 3B–3E).

To explore whether the up-regulation of ANGPTL4 was related to the increased expression of TGF-β2, we further investigated the level of ANGPTL4 in GCTSCs under the treatment of TGF-β2 (Figure 3F). The results indicated that TGF-β2 increases the expression level of ANGPTL4 in GCTSCs in a time-dependent way. In addition, we treated GCTSCs and BMSCs with TGF-β2 and its corresponding inhibitor LY2109761, then, measured the protein levels of ANGPTL4 in each sample, respectively (Figure 3G). Western blot assay revealed that LY2109761 could down-regulate the endogenous ANGPTL4 level. Moreover, we cloned the putative human ANGPTL4 promoter in a luciferase reporter. The luciferase reporter assay indicated that Smad3, Smad4 and HIF-1α could directly activate the promoter of ANGPTL4. To further determine the regulation of ANGPTL4 by these TFs, we performed chromatin immunoprecipitation (ChIP) analysis. The results revealed that all the three TFs could interacted with the promoter of ANGPTL4 (Supplementary Figure 1B).

Taken together, these results suggested that a high level of TGF-β2 could contribute to the increased ANGPTL4 expression in GCTSCs.

### ANGPTL4 regulates multinucleated cell formation and angiogenesis *in vitro*

**Figure 4:**
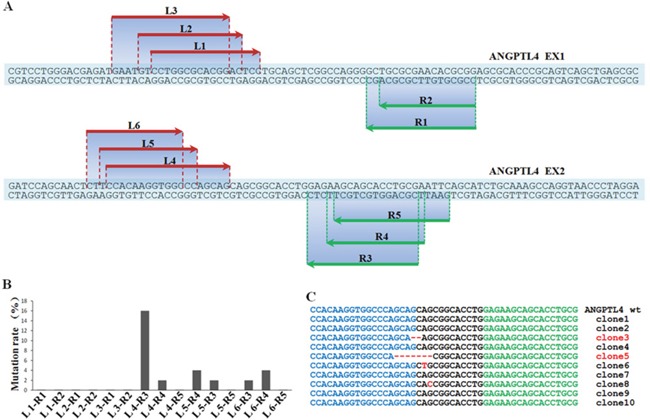
Detailed experimental schematic diagram of ANGPTL4 knockout in GCTSCs (GCTSCsANG-/-) using TALENs method

In order to reach a more comprehensive understanding of the regulatory role of ANGPTL4 in GCT, we established a stable GCTSCs cell line with *ANGPTL4* deletion (GCTSCs^ANG-/-^) using TALENs method. Detailed experimental schematic diagram was shown in Figure 4.

**Figure 5:**
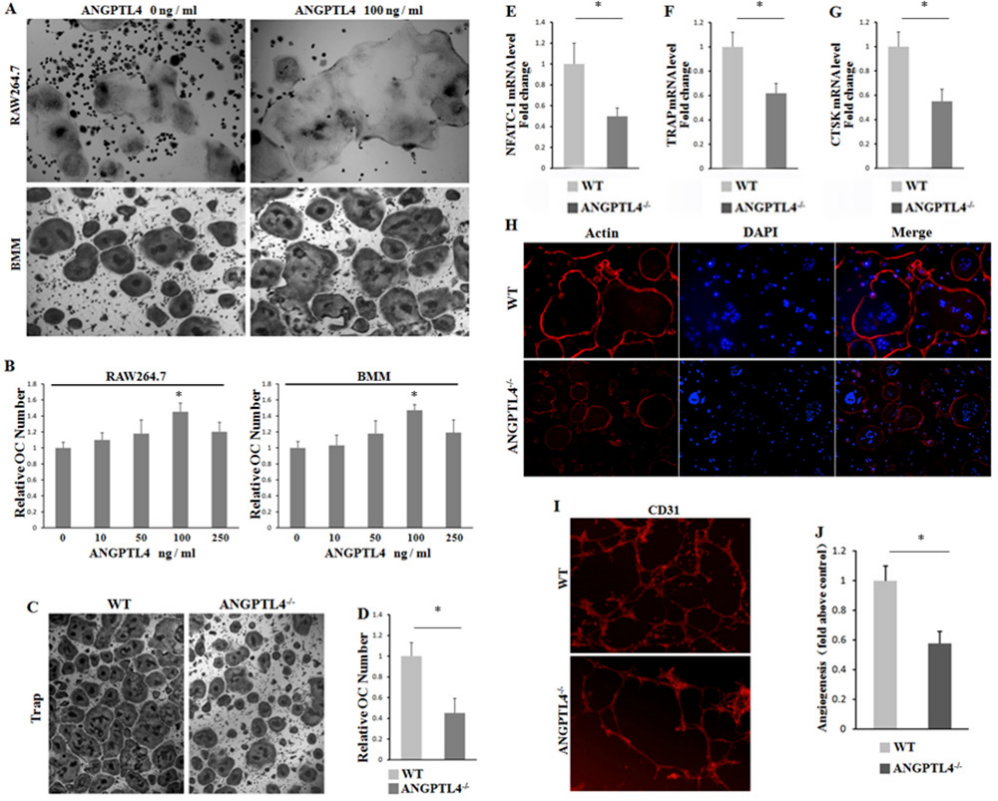
ANGPTL4 promotes GCTSC induced osteoclast proliferation and angiogenesis *in vitro* **(A** and **B)** RAW264.7 and BMM cells were treated with indicated concentration of ANGPTL4 for 5 days. The numbers of multinucleated osteoclasts were determined by TRAP staining **(A)** and the numbers of TRAP-positive multinucleated (>5 nuclei) osteoclasts were counted as treated with indicated concentration of ANGPTL4 in RAW264.7 and BMM cells **(B)**. **(C)** TRAP staining of BMM cells cultured with conditional mediums of GCTSCs^ANG-/-^ and GCTSCs^WT^, respectively. **(D)** the numbers of TRAP-positive multinucleated (>5 nuclei) osteoclasts were counted. **(E-G)** qRT-PCR analysis of NFATC1, TRAP and CTSK expression in BMM cells. **(H)** rhodamine phalloidin staining of BMM cells from GCTSCs^ANG-/-^ and GCTSCs^WT^ group, respectively. **(I** and **J)** CD31 staining of BMM cells and analyzing the number of vessel sprouting, * *p*<0.05.

To quest the biologic role of ANGPLT4 during regulating the multinucleated cell formation in GCT, we used RAW264.7 and BMM cells with M-CSF stimulation as osteoclast (OC) differentiation model *in vitro*. Two groups of RAW264.7 and BMM cells were treated with ANGPTL4 (0 ng/ml) and ANGPTL4 (100 ng/ml), respectivlely, then cultured with conditional mediums M-CFS (10ng/ml) for 3 days. Our results showed that ANGPTL4 could dramatically promote multinucleated cell formation in both RAW264.7 and BMMs cells (Figure 5A and 5B). In addition, we also found that culturing with GCTSCs^ANG-/-^ conditional medium markedly inhibited BMM differentiation by TRAP staining and actin-ring formation assay (Figure 5C and 5H). The results of qRT-PCR analysis of NFATC1, TRAP and CTSK expression in BMM cells further confirmed the BMM differentiation (Figure 5E–5G). To explore the angiogenesis of ANGPTL4, we cultured HUVEC with GCTSCs^ANG-/-^ and GCTSCs^WT^ conditional mediums respectively for 18 hours. Strikingly, angiogenic ability was much lower in the group with GCTSCs^ANG-/-^ conditional mediums than in the controls (Figure 5I and 5J).

**Figure 6:**
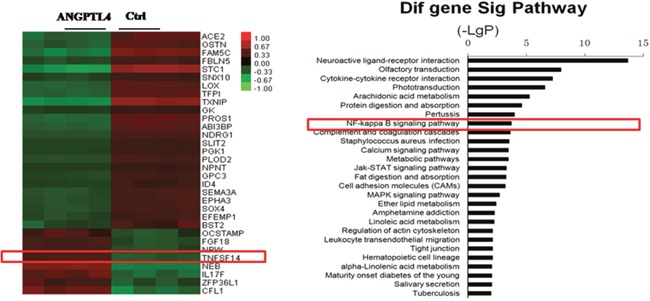
Heat map and pathway analysis of differentially expressed genes in GCTSCs stimulated with or without recombinant ANGPTL4 Right: Microarray assays showed bone and tumor metabolism asscociated genens in recombinant ANGPTL4 stimulation group compared with control group. (Red box denoted the gene TNFSF14 which could increases osteoclastogenesis and decreases osteoblastogenesis) Left: Patheway analysis showed in GCTSCs stimulated with or without ANGPTL4-AP. (Red box denoted NF-kappa B signaling pathway which could activate mature osteoclasts.)

To gain further insights into the mechanism of ANGPTL4 in GCT of bone, we performed microarray assay in GCTSCs stimulated with or without recombinant ANGPTL4. On the basis of these results, we found 32 differently expressed genes associated with bone and tumor metabolism (Supplementary Table 3). Among these genes, we verified the six most closely related to bone metabolism, which was consistent with the results of microarray (Supplementary Figure 1C). In these genes, we focused on one gene named TNFSF14, which was significantly increased in recombinant ANGPTL4 stimulation GCTSCs. Some studies indicated that this gene was in association with the increase of osteoclastogenesis and the decrease of osteoblastogenesis [32]. We also performed pathway analysis of these related genes and found that NF-kappa B pathway is significantly involved. In this pathway, RANKL binding to its receptor NF-kappa B (RANK) provided the crucial signal to drive osteoclast development from haematopoietic progenitor cells as well as to activate mature osteoclasts. (Figure 6)

Collectively, these results suggest that ANGPTL4 regulates osteoclast differentiation and promotes angiogenesis *in vitro*.

### ANGPTL4 promotes cell proliferation and angiogenesis of GCT *in vivo*

**Figure 7:**
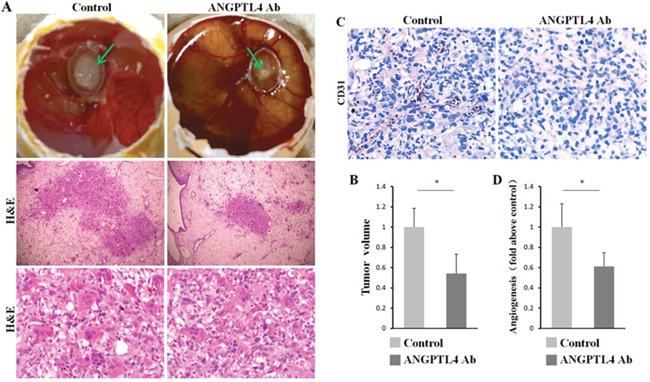
ANGPTL4 increases tumor proliferation and angiogenesis *in vivo* **(A)** the tumor solute was seed into the plastic ring on the CAM with condition mediums of ANGPTL4 inhibitor or not, after 6 days of incubation. **(B)** the analysis of tumor sizes in two groups. **(C** and **D)** the CD31 staining and the assay of angiogenesis of tumor in two groups, * *p*<0.05.

**Figure 8:**
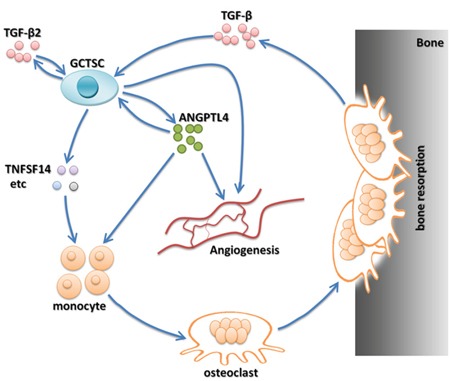
ANGPTL4 acting as a critical cytokine modulates the biological cycle formation of GCT-bone microenvironment

To validate our results *in vivo*, first, we successfully established the chick CAM assay for human GCT of bone. After 10 days of incubation, 20 μl of re-suspended GCT suspension were deposited into two groups of plastic rings in the CAM with or without ANGPTL4 inhibitor. Then, the tumor size and angiogenic activity were measured after another 6 days of incubation. As expected, ANGPTL4 inhibitor could strikingly decreased the proliferation of GCT, as well as the tumor volume in xx model (Figure 7A and 7B). Moreover, CAM model of GCT treated with ANGPTL4 inhibitor exhibited less CD31 staining (Figure 7C), suggesting that inhibition of ANGPTL4 attenuated the angiogenic ability of GCT (Figure 7D). Taken together, these data suggested that ANGPTL4 regulates GCT progression and acts as a critical cytokine modulating the biological cycle formation of GCT-bone microenvironment through TGF-β signalling. (Figure 8)

## DISCUSSION

GCT is a locally aggressive and highly osteolytic bone tumor [2]. Abundant osetolysis and GCTSCs proliferation are the most prominent characters of GCT which bring great distress to the patients and pose challenges in treatment [1]. In addition, our previous study has showed that inhibition of bone resorption could strikingly reduce the post-operative recurrence rate of GCT [2, 9]. Control of tumor proliferation and intervention to reduce osetolysis formation are primary directions in research and treatment of GCT, however, the mechanism has hitherto not fully defined. Here, we demonstrated that ANGPTL4 was secreted at a high level by GCTSCs in GCT and could be regulated by TGF-β2 through TGF-Smad signaling pathway. By knocking–out *ANGPTL4* using TALENs method, osteoclastogenesis, osteolysis and angiogenesis induced by GCTSCs were significantly suppressed *in vitro*. Furthermore, by establishing the chick CAM model of human GCT, we found that ANGPTL4 could promote the proliferation and angiogenesis of GCT *in vivo*. In addition, some new downstream genes of ANGPTL4 were identified through microarray assay. Therefore, the present results suggest that ANGPTL4 promoted GCTSC induced osteoclast proliferation and angiogenesis *in vitro* and *in vivo*.

The previous studies have showed that ANGPTL4 was mainly expressed in liver, adipose tissue, placenta and ischemic tissues [15, 16, 22]. It was directly involved in regulating glucose homeostasis, lipid metabolism, and insulin sensitivity. It was demonstrated to be a new member of the angiopoietin family of regulators, and independently to be targets of PPAR family of metabolic response transcription factors [30, 33]. Several researches have indicated that HIF-1α could strongly enhance ANGPTL4 mRNA expression level in response to hypoxia [28, 34]. The evidence of one research has supported the role for ANGPTL4 enhanced by HIF-1α stimulation of osteoclast resorptive activity [34]. However, in our study the expression of FOXO1, HIF-1α, PPAR-α and PPAR-γ was no significant difference in GCTSCs compared to BMSCs, indicating that the high level of ANGPTL4 may not be regulated by above-mentioned transcription factors.

TGF-β is a multifunctional cytokine having diverse effects on almost all cell types and playing pivotal roles during embryo development and tissue homeostasis [21, 35]. It was widely reported that this cytokine could regulate the production of microenvironment sensors and modulators, including cytokines, extracellular matrix components, and cell-surface receptors [31, 35]. Additionally, with the tumor microenvironment, TGF-β could play as a natural response to the hypoxic and inflammatory conditions that occur during tumor progression [36]. Thiolloy et al. [37] confirmed that the mechanism involving the activation of latent TGF-β was critical for the metastatic tumor survival in the osteolytic tumor-bone microenvironment. Recently, Padua et al. have demonstrated that it is central to the process of breast cancer cells metastasis to the lungs through TGF-β induction of ANGPTL4 via the Smad signaling pathway [21]. In the present study, we found that TGF-β was highly expressed in GCT and ANGPTL4 was regulated by TGF-β at a time-dependent way. Furthermore, we also confirmed that it was the member TGF-β2 in the TGF-β family playing the role in GCTSCs and then promoting bone resorption.

Although the primary known roles of ANGPTL4 to date are in regulation of lipid metabolism and angiopoiesis, Knowles et al. [38] have reported that at high local concentrations ANGPTL4 could stimulate osteoclast activity. In addition, ANGPTL4-mediated induction of osteoclast activity could be achieved in the absence of RANKL [38, 39]. In this study, we found that ANGPTL4 was overexpressed in GCT compared with that in the normal tissue. We further detected that the expression of ANGPTL4 was secreted by GCTSCs which was considered as the only neoplastic component. Moreover, the knockout of ANGPTL4 could significantly attenuate the effect of GCTSC proliferation and tumor angiogenesis. Furthermore, the result of our microarray assay showed that several cytokines were involved in bone and tumor metabolism. The downstream target genes of ANGPTL4 were up-regulated after the activation of ANGPTL4 in GCTSCs, including but not limited to STC1, TXNIP, FAM5C, PROS1 and ACE2. Among these downstreams of ANGPLT4, it was interesting to find that TNFSF14 was recognized in this study. This cytokine was reported to have the effect of increasing osteoclastogenesis and decreasing osteoblastogenesis in multiple myeloma-bone disease [32]. Functional analysis indicated that NF-kappa B signaling pathway was significantly involved in the results of our microarray assay. The discovery of the factors involved in the control of osteoclasts has moved bone research into a new era. These factors are the receptor activator of NF-kappa B (RANK), its ligand RANKL and the decoy receptor for RANKL, osteoprotegerin (OPG) [40]. Binding of RANKL to RANK triggers intricate and distinct signaling cascades to induce osteoclast development from haematopoietic progenitor cells as well as to activate mature osteoclasts [41]. Meanwhile, OPG negatively regulates RANKL binding to RANK and therefore inhibits bone turnover by osteoclasts [42]. Several researches demonstrated that stimulation of RANK results in strong NF-kappa B activation and the NF-kappa B signaling pathway is relevant for RANKL-RANK-regulated osteoclast development and function [41, 42]. These results indicate that ANGPLT4 might promote GCT progression by activating GCTSC proliferation and angiogenesis through the regulation of a series of downstream cytokines.

The role of ANGPTL4 involved in the regulation of angiogenesis remains controversial [15, 22, 23]. It has been demonstrated that ANGPTL4 could inhibit VEGF-induced cell proliferation, migration, and tubule formation in HUVECs and this effect was via the suppression of Raf/MEK/ERK signaling pathway. Furthermore, a recent study has found that tumor-induced ANGPLT4 could inhibit vascular tube formation and proliferation of HUVECs *in vitro*, mainly through the MEK pathway [43]. However, Perdiguero et al. [44] have demonstrated that ANGPTL4 modulates sprouting, branching and maturation of the retinal vascular plexus. Besides, ANGPTL4 is highly expressed in retinal vascular plexus induced by hypoxia [22]. Thus, the function of ANGPTL4 appears to be tissue dependent. In our study, we demonstrated that ANGPLT4 may significantly promote angiogenic ability of GCT *in vitro and vivo*, to stimulate tumor growth. Currently, ANGPLT4 is considered to be an orphan ligand because information regarding its potential binding partner is lacking. In this regard, it is necessary to find ANGPTL4 corresponding receptors to have a better understating of the underlying mechanisms in angiogenesis.

In conclusion, our study initially demonstrated that ANGPLT4 was up-regulated in GCT of bone, and highly expressed TGF-β could stimulate ANGPLT4 in GCTSCs. Furthermore, ANGPLT4 played a critical role in regulating tumor cell proliferation and angiogenesis, possibly by regulating STC1, EPHA3 and TNFSF14 genes. In addition, knockout of ANGPLT4 also had an inhibitory effect on giant cell formation both and angiogenesis *in vitro and in vivo*. Taken together, our study for the first time identified the role of ANGPLT4 in GCT of bone, which may provide a new possible target for the diagnosis and treatment of GCT.

## MATERIALS AND METHODS

### Clinical samples and cell culture

The study population comprised 30 Chinese GCT patients with the age range from 21 to 56 years who underwent surgical resection for primary GCT in our department between September 2012 and June 2014 (Supplementary Table 1). Patients who received radiotherapy/chemotherapy or had co-existing diabetes mellitus, hematologic diseases and rheumatoid arthritis that may affect bone metabolism were excluded from the analysis. The para-tumor tissues from the same GCT patients were used as normal controls. Samples were snap-frozen and stored in liquid nitrogen within two hours after surgical excision.

This study was approved by the Ethnic Committee of Shanghai Changzheng Hospital of the Second Military Medical University (Shanghai, China), and written informed consent was obtained from all participants.

### Cell culture

For primary cell culture, bone marrow macrophages (BMMs) isolated from C57/BL6 mice and GCTSCs isolated from GCT samples were cultured as described previously [24]. Bone marrow mesenchymal stem cells (BMSCs) were isolated from the bone marrow of the GCT patients as described previously [25]. BMMs and GCTSCs were maintained in α-MEM supplemented with 10% fetal bovine serum (FBS) in the cell incubator (37 °C, 5% CO_2_). BMSCs were cultured in DMEM/F-12 supplemented with 10% FBS. Human umbilical vein endothelial cells (HUVECs) and HEK293 CELLS were purchased from the American Type Culture Collection (Manassas, VA, USA) and cultured in EGM-2 and DMEM with 10% FBS, respectively.

### qRT-PCR

Total RNA was isolated by using TRIZOL (Invitrogen, Carlsbad, CA, USA) and reverse transcribed into cDNA by using the PrimeScript™ RT Master Mix (Takara, Shiga, Japan). Gene transcripts were quantified on 7900HT Fast Real-Time PCR System (Life Technologies Corporation, Carlsbad, California, USA) using SYBR® Premix Ex Taq™ II (Takara). All primers were listed in Supplementary Table 2.

### Immunohistochemical (IHC) staining

The tissue sections were stained with hematoxylineosin and by IHC using indirect immunoperoxidase technique. The antibodies of ANGPTL4 (ab115798) was purchased from Abcam (Cambridge, UK), and the antibodies of Foxo1 (sc-34890), HIF-1α (sc-13515), PPAR-α(sc-130640), PPAR-γ(sc-81152), TGF-β1(sc-52892), TGF-β2(sc-374658), TGF-β3(sc-166833) and CD31(sc-13537) were brought from Santa (Santa Cruz, CA, USA).

### Western blot

Western blot assay for ANGPTL4, Foxo1, HIF-1α, PPAR-α, PPAR-γ, TGF-β1, TGF-β2 and TGF-β3 was performed using the antibodies described in IHC staining. Beta-actin protein was determined using the specific antibody (Santa) as a loading control.

### Immunofluorescence (IF) staining

IF staining was performed using the antibody of ANGPTL4 (Abcam) following the protocol reported previously [26]. The signals of IF were examined with a BX51 fluorescence microscope (Olympus, Tokyo, Japan).

### Enzyme-linked immunosorbent assay (ELISA)

The levels of ANGPTL4 in serum were detected by ELISA kits (Abcam) according to the manufacturer's instruction. Serum was diluted with the sample dilution buffer with a ratio of 1:1. Standard curves were created using purified ANGPTL4 and the CurveExpert 1.4 software program.

### Luciferase reporter assay and chromatin-immunoprecipitation (ChIP) assay

pGL3-basic plasmid was enzyme digested by KpnI and XhoI (TransGen Biotech, Beijing, China). The possible promoter region of ANGPTL4 (-2000 ˜ 0 bp from the initiation codon) was synthesized by GENEWIZ Inc (Suzhou, China) and inserted into pGL3-basic by using the Quick-Fusion Cloning Kit (Biotool, Houston, TX, USA). The overexpression (OE) plasmids of Smad3, Smad4 and HIF-1α were purchased from Vigene Biosciences (Jinan, China). Dual luciferase assays were conducted in a 24 well plate format. pGL3-ANGPLT4-promoter report + TK100 Renilla report were transfected into 60% confluent HEK293 cells, along with OE-Smad3 or OE-Smad4 plasmids or OE-HIF-1α or negative control. After 48 h transfection, firefly and renilla luciferase were quantified sequentially using the Dual Luciferase Assay kit (Promega, Madison, WI, USA) following the manufacturer's recommendations.

Chromatin-immunoprecipitation (ChIP) analysis was performed using a ChIP Assay Kit (Cell Signaling Technology) according to the manufacturer's protocols. The immunoprecipitated and input DNA were used as templates for PCR analysis, and the primers are listed in Supplementary Table 2.

### Construction of stable knockout cell lines using TALENs

The plasmids of TALENs for ANGPTL4 gene were designed and constructed using Fast TALETM TALEN Assembly kit from SiDanSai biotechnology (Shanghai, China). To validate the activities of synthesized endonucleases, an equal amount of expression vectors for the left and right TALENs or sgRNA were transferred into HEK293 cells. The most activated pair of TALENs was transfected into GCTSCs. After selection with puromycin, resistant colonies were picked up and examined by genomic PCR and gene sequencing.

### Tube formation assay

50 μl of chilled Matrigel (BD Bioscience, Franklin Lakes, NJ, USA) was added to a 96-well plate and incubated at 37 °C for 30 min. HUVECs (1 × 10^4^ cells) were suspended in 100 μl of EBM-2 and conditioning medium from GCTSCs or ANGPLT4 knockout GCTSCs, and added to the solidified Matrigel. After 18h incubation, angiogenesis was assessed on the basis of capillary-like structure formation. Tubes in randomly chosen five microscopic fields were photographed and counted.

### TRAP staining and actin ring formation

For TRAP staining, cells were fixed and stained using the TRAP staining kit (Sigma). TRAP-positive multinucleated cells containing three or more nuclei were counted as mature osteoclasts. For actin ring formation assay, cultured BMMs were first fixed with 4% PFA in PBS for 10 min, permeabilized with 0.1% Triton-X 100 in PBS for 5 min, and then incubated with rhodamine-conjugated phalloidin (Molecular Probes, Eugene, OR, USA). The ring structure of F-actin dots that indicates osteoclastogenesis was observed under a fluorescence microscope.

### Chick chorio-allantoic membrane (CAM) model construction

A short-term model of GCT in vivo was set up in the chick CAM as described previously [4]. BrefFertilized white leghorn chicken eggs (Valo-SPF eggs, LohmannTierzucht GmbH, Cuxhaven, Germany) were incubated at a humidity of 70% and 37°C. At embryonic day 3, 2-3ml albumen was removed with a syringe. After 10-day incubation, small plastic rings made of Thermanox™ cover discs were placed on the CAM. After gentle laceration of the CAM surface, 20 μl re-suspended tumor suspension with 2ug ANGPLT4 antibodies (ANGPLT4 Ab, Abcam) or PBS was deposited into the rings. ANGPLT4 antibodies or PBS was injected additionally at day 13. Until day 16, CAMs were measured and collected for further analysis. All embryos that died before day 16 were excluded from further analysis.

### Microarray assay

Total RNA was extracted from GCTSCs using TRIZOL (Invitrogen). The concentration of the RNA was analyzed using the NanoDrop2000 (Thermo Scientific, Wilmington, MA, USA). Microarrays were performed by utilizing the GeneChip® Human Transcriptome Array 2.0 (Affymetrix, Santa Clara, CA, USA) according to the manufacturer's instructions. Raw data were corrected by background subtraction, and then normalized within and between each array. The differentially expressed genes and alternatively spliced genes were analyzed by Affymetrix Transcriptome Analysis Console software. Statistical comparisons were performed by analysis of variance.

### Statistics

SPSS 19.0 statistical software (SPSS Inc, Chicago, IL, USA) was used for statistical analysis. All data are presented as mean ± standard error of the mean. The comparison of IHC staining between groups was performed by Chi-square test. Statistics of the mean value between groups were assessed using independent Student t test, assuming double-sided independent variance. All experiments were repeated at least three times, and representative experiments are shown. *P* values ≤ 0.05 were considered statistically significant.

## SUPPLEMENTARY MATERIALS FIGURES AND TABLES


