# Supplementary material for: TGF-β2-induced ANGPTL4 expression promotes tumor progression and osteoclast differentiation in giant cell tumor of bone
Source: Oncotarget. 2017 Jun 27;8(33):54966–77. doi: 10.18632/oncotarget.18629 (PMC5589634; doi:10.18632/oncotarget.18629)
Supplement: Supplementary file 1 [file oncotarget-08-54966-s001.pdf]

TGF-β2-induced ANGPTL4 expression promotes tumor progression and osteoclast differentiation in giant cell tumor of bone

SUPPLEMENTARY MATERIALS

SUPPLEMENTARY FIGURE AND TABLES

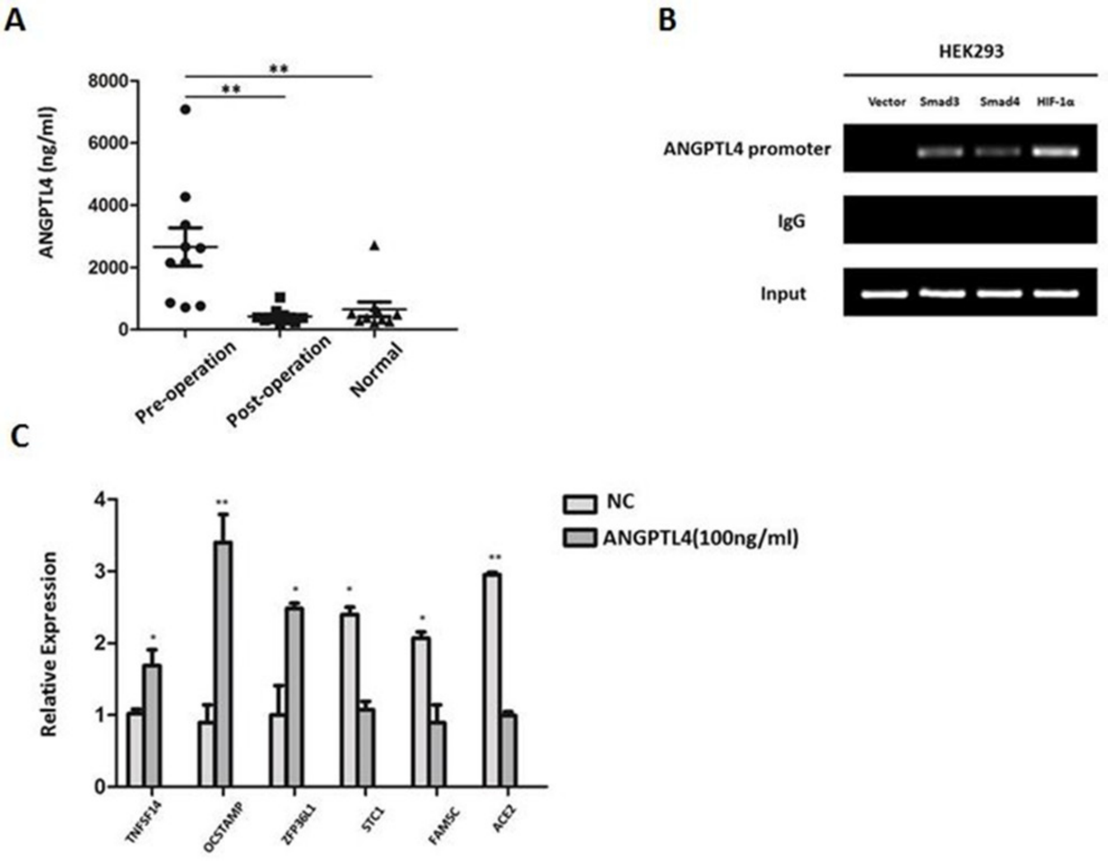

Supplementary Figure 1: (A) ELISA assay of secreted ANGPTL4 in serum in different groups. Pre-operation group: in patients with GCT confirmed before operation. Post-operation group: postoperative patients without obvious recurrence at least 6 months after operation. Normal group: in patients without GCT or hepatic disease. Each group included 10 patients. **(B)** identification of Smad3, Smad4 and HIF-1a binding to ANGPTL4 promoter *in vitro* by CHIP assay. Lysates from HEK293 cells were subjected to ChIP by using ChIP assay kit from CST. Sonicated chromatin was used as input and IgG antibody was used as negative control. **(C)** mRNA levels of differentially expressed genes closely related to bone metabolism in GCTSCs stimulated with or without recombinant ANGPTL4, * $p<0.05$, ** $p<0.01$.

Supplementary Table 1: Characteristics of the 30 GCT patients in this experiment

Factors	GCT of bone patients
Age (years)	35.3±10.2
Sex (male/femal)	12/18
Disease history (months)	22.6±21.8
Tumor size (cm)	7.2±2.7
Tumor site (spine/limbs)	28/2
Primary/recurrence	26/4
Resection (total/subtotal)	28/2

Supplementary Table 2: Primers for qRT-PCR assays and CHIP assays

Gene symbols	Forward	Reverse	Product size(bp)
human TGF-β1	GGAGCGGAGGAAGGAGTC	AAAGGGAGGCGGTCTGG	247
human TGF-β2	CCTCTAACCATTCTCTACTACATTG	TCGTCGTCATCATCATTATCATC	148
human TGF-β3	GGCGTGGACAATGAGGATG	AGCAGAAGTTGGCATAGTAGC	280
mouse NFATC1	CTCACCACAGGGCTCACTA	GATGGCTCGCATGTTATTT	284
mouse TRAP	GCCCTTACTACCGTTTGC	TCTCGTCCTGAAGATACTGC	351
mouse CTSK	AGTAGCCACGCTTCCTAT	CATCCACCTTGCTGTTAT	182
human ANGPTL4	GACCTCCGCAGGGACAAGA	TCGGGCAGAGCCAAGAGT	281
human ANGPTL4 promoter	GGCTGGTCTGGAAGTCTTG	GGGCGGAGGAGTCTTGG	309
human FOXO1	AACAGCCACCACTCTATCATC	GCACCAAGTTCAGTTACATACC	111
human HIF-1α	TAGCCGAGGAAGAACTATGAAC	ACTGAGGTTGGTTACTGTTGG	101
human PPAR-α	TGGCTCCTGAAATGACTCTAAG	GATGTCTGGTGCTGGTTCC	122
human PPAR-γ	TTACGAAGACATTCCATTCACAAG	TATCTCCACAGACACGACATTC	203
human TNFSF14	CTTCTTGCTGTTGTTCATTGC	GGATGCTTCATTCGCTTGG	106
human OCSTAMP	ACGCTCACGGTCAAGTATG	TGTGGACGGAGAGGAAGG	100
human ZFP36L1	CCGCACACTTATTCCTCCTC	GGTCTGGCAACAACTCTTCC	104
human STC1	GAGGCGGAGCAGAATGAC	CGAATGGCGAGGAAGACC	278
human FAN5C	CTCTTGCTTGCTTCGTCATAG	GGTCGTGTCATAATCCATTGTG	115
human ACE2	GAGGAGGATGTGCGAGTG	CAGGATGACAATGCCAACC	282
human GAPDH	GGAGTCCACTGGCGTCTTCA	GGGGTGCTAAGCAGTTGGTG	191
mouse GAPDH	TGTTTCCTCGTCCCGTAG	CAATCTCCACTTTGCCACT	108

Supplementary Table 3: Part of the possible downstreams of ANGPTL4 in GCTSCs that might function in tumor progression and osteolysis

Gene symbols	Gene name	Main function in bone metabolism	Fold change*
STC1	stanniocalcin 1	Inhibit osteoclast activity	0.43
TXNIP	thioredoxin interacting protein	Treating osteoclasts with silenced TXNIP OB media showed an increased osteoclast activity	0.45
FAM5C	Family with sequence similarity 5, member C	Enhance osteoblast differentiation in differentiated osteoblasts	0.5
PROS1	protein S (alpha)	Deficiency of protein-S secretion by the osteoblasts contributes to osteopenia	0.55
ACE2	angiotensin I converting enzyme 2	Promote osteoclastogenesis	0.56
ABI3BP	ABI family, member 3 (NESH) binding protein	Displaye severe deficiencies in osteogenic differentiation after knockout	0.57
TFPI	tissue factor pathway inhibitor	Inhibition of angiogenesis	0.57
LOX	lysyl oxidase	Promote osteoblast proliferation	0.57
OSTN	osteocrin	Modulate the osteoblast phenotype	0.58
NDRG1	N-myc downstream regulated 1	Inhibition of angiogenesis	0.62
PLOD2	procollagen-lysine, 2-oxoglutarate 5-dioxygenase 2	Resembling osteogenesis imperfecta due to a recessive mutation	0.62
SEMA3A	semaphorin 3A	Regulate bone remodelling indirectly by modulating sensory nerve development	0.62
EPHA3	EPH receptor A3	Inhibition of angiogenesis	0.62
SOX4	SRY (sex determining region Y)-box 4	Exhibit significantly lower bone mass in Sox4(+/-) mice	0.63
FBLN5	fibulin 5	Interfere with choroidal neovascularization by downregulating VEGF	0.64
EFEMP1	EGF containing fibulin-like extracellular matrix protein 1	Inhibition of angiogenesis	0.64
BST2	bone marrow stromal cell antigen 2	Mediate monocyte adhesion to human endothelial cells by IFN-γ	0.64
GPC3	glypican 3	Decrease osteoclast differentiation in glypican-3-deficient mice	0.65
SLIT2	slit guidance ligand 2	Regulate *in vitro* osteoblast differentiation	0.66
SNX10	sorting nexin 10	Related to osteoclast differentiation *in vitro* and expressed in osteoclasts *in vivo*	0.66
PGK1	phosphoglycerate kinase 1	Regulates bone formation at the metastatic site by increasing osteoblastic activity	0.67
ID4	inhibitor of DNA binding 4	Promote osteoblast and adipocyte differentiation	0.67
NPNT	nephronectin	Promote osteoblast differentiation	0.68
GK	glycerol kinase	Moderates osteoporosis with the deficiency of glycerol kinase	0.68
OCSTAMP	osteoclast stimulatory transmembrane protein	Regulate osteoclast proliferation	1.41
FGF18	fibroblast growth factor 18	Accelerate osteoblast differentiation by upregulating Bmp2 expression.	1.43
NPW	neuropeptide W	Regulate feeding behavior	1.43
ZFP36L1	ZFP36 ring finger protein-like 1	Regulate angiogenesis	1.46
TNFSF14	tumor necrosis factor (ligand) superfamily, member 14	Increase osteoclastogenesis and decreases osteoblastogenesis in multiple myeloma-bone disease	1.5
IL17F	interleukin 17F	Regulate rheumatoid arthritis inflammation and joint destruction	1.63
CFL1	cofilin 1	In association with angiogenesis	1.75
NEB	nebulin	A giant protein component of the cytoskeletal matrix	1.88

*means the fold change of the selected genes in microarray assay results
